# Reprogramming Glia Into Neurons in the Peripheral Auditory System as a Solution for Sensorineural Hearing Loss: Lessons From the Central Nervous System

**DOI:** 10.3389/fnmol.2018.00077

**Published:** 2018-03-14

**Authors:** Steven J. Meas, Chun-Li Zhang, Alain Dabdoub

**Affiliations:** ^1^Department of Laboratory Medicine and Pathobiology, University of Toronto, Toronto, ON, Canada; ^2^Biological Sciences, Sunnybrook Research Institute, Toronto, ON, Canada; ^3^Department of Molecular Biology, The University of Texas Southwestern Medical Center, Dallas, TX, United States; ^4^Department of Otolaryngology – Head & Neck Surgery, University of Toronto, Toronto, ON, Canada

**Keywords:** brain, ear, hearing loss, *in vivo*, regeneration, reprogramming, tissue repair

## Abstract

Disabling hearing loss affects over 5% of the world’s population and impacts the lives of individuals from all age groups. Within the next three decades, the worldwide incidence of hearing impairment is expected to double. Since a leading cause of hearing loss is the degeneration of primary auditory neurons (PANs), the sensory neurons of the auditory system that receive input from mechanosensory hair cells in the cochlea, it may be possible to restore hearing by regenerating PANs. A direct reprogramming approach can be used to convert the resident spiral ganglion glial cells into induced neurons to restore hearing. This review summarizes recent advances in reprogramming glia in the CNS to suggest future steps for regenerating the peripheral auditory system. In the coming years, direct reprogramming of spiral ganglion glial cells has the potential to become one of the leading biological strategies to treat hearing impairment.

## Introduction

It is estimated that disabling hearing loss affects 360 million people worldwide, which is over 5% of the world’s population (Olusanya et al., 2014; World Health Organization, 2015). This makes hearing loss the most prevalent form of sensory impairment (Gaylor et al., 2013; Müller and Barr-Gillespie, 2015). Hearing disability is also widespread across all age groups; 0.3% of newborns, 5% of people by the age of 45 and 50% of people by the age of 70 experience some form of congenital or acquired hearing loss (Kral and O’Donoghue, 2010; Sprinzl and Riechelmann, 2010). Many individuals suffering from impaired hearing also experience a significant decrease in quality of life and are more likely to suffer from depression (Mulrow et al., 1990). Therefore, there is a pressing need to discover new strategies to repair hearing.

**FIGURE 1:**
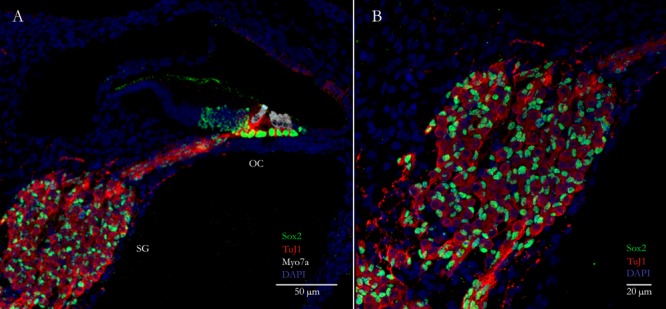
Primary auditory neurons (PANs) innervate sensory hair cells in the cochlea. **(A)** Cross-section through a neonatal mouse cochlea showing spiral ganglion glial cells labeled with Sox2 (green nuclei) surrounding PANs labeled with TuJ1 (red) in the spiral ganglion (SG). PANs innervate mechanosensory hair cells labeled with the specific hair cell marker Myosin7a (white) in the organ of Corti (OC). **(B)** Higher magnification image of the spiral ganglion showing the PANs (red) and surrounding glial cells (green).

The auditory system works by converting sound waves into electrical signals that are transmitted to the brain. The tympanic membrane at the end of the external ear canal conveys vibrations in the air to the small bones, or ossicles, of the middle ear. These vibrations are conducted through the ossicles and passed onto the oval window, which separates the middle and inner ears. The movement of the oval window causes disturbances in the fluid of the cochlear duct and these fluctuations are detected by mechanosensory hair cells in the organ of Corti, which transform this information into chemical signals received by the dendrites of primary auditory neurons (PANs) that emerge from the spiral ganglion (**Figure 1**). The hair cells of the organ of Corti form one row of inner hair cells followed by three rows of outer hair cells. Inner hair cells are innervated by Type I PANs, which compose 90–95% of PANs, are large and myelinated whereas outer hair cells are innervated by Type II PANs, which compose 5–10% of PANs, are small and unmyelinated (Nayagam et al., 2011). Type I afferents are the primary receptors for auditory signaling. Unfortunately, less is known regarding Type II function, however, it appears strong acoustic stimulation is required for activation (Weisz et al., 2009). These glutamatergic PANs relay an electrical impulse from the cochlea, the sensory organ for hearing, to the auditory centers in the brain through the eighth cranial nerve (Appler and Goodrich, 2011). There are two primary categories of hearing loss based on the location of pathology: conductive and sensorineural. The former includes forms of impairment in conveying sound waves through the outer or middle ear. The latter includes forms of impairment resulting from damage to the components of the cochlea, including hair cells and/or PANs (Liberman, 2017). Sensorineural hearing loss can manifest after viral infection, exposure to otherwise lifesaving ototoxic drugs, noise and/or aging (White et al., 2000; Kral and O’Donoghue, 2010; Olusanya et al., 2014; Ruan et al., 2014; Liberman, 2017). Traditionally it was thought that PANs could only become damaged as a result of hair cell loss; a form of PAN damage known as secondary degeneration (Bohne and Harding, 2000; McFadden et al., 2004; Stankovic et al., 2004; Sugawara et al., 2005). However, it is now understood that PAN loss can occur independent of damage to hair cells; a form of PAN damage known as primary degeneration (Kujawa and Liberman, 2006; Lin et al., 2011; Makary et al., 2011). The primary degeneration of PANs leads to a condition known as auditory neuropathy, where the mechanosensory hair cells of the cochlea remain intact but PANs are lost. Primary degeneration can develop as a consequence of glutamate excitotoxicity (Zheng et al., 1997), noise exposure (Lin et al., 2011; Furman et al., 2013), and/or genetic defects (Angeli et al., 2012). This type of sensorineural damage, is one of the leading features of presbycusis, or age-related hearing loss, and is characterized by difficulty hearing in noisy settings (Kujawa and Liberman, 2015). In fact, although presbycusis can present itself through four pathological categories; sensory, neural, metabolic and mechanical, where metabolic refers to degeneration of the stria vascularis and mechanical refers to hardening of cochlear membranes. Neuronal loss is characterized as the best indicator for age-related hearing degeneration (Schuknecht and Gacek, 1993).

Once PANs are lost they will never regenerate, hence regenerative medicine techniques hold enormous potential for the recovery of PANs in the spiral ganglion. This is especially significant considering that modern clinical solutions for hearing impairment rely solely on medical devices such as hearing aids and cochlear implants (Müller and Barr-Gillespie, 2015). These assistive technologies have provided a much-needed boon to the lives of patients, however, they are only suitable for a limited population of hearing impaired individuals and even when compatible do not resemble natural hearing or make music enjoyable, as reported by users (Briggs, 2011). One of the main factors involved in the effectiveness of cochlear implants is the health and numbers of PANs (Yagi et al., 2000). Hence, to improve the quality of life for individuals suffering from hearing impairment there needs to be new interventions that (1) address the population where current devices are not appropriate and (2) improve the quality of hearing toward a natural level. Biological strategies currently being investigated to replace and/or protect PANs include stem cell (Nayagam et al., 2013) and growth factor therapies (Gillespie et al., 2014; Müller and Barr-Gillespie, 2015). Another option to consider is the direct reprogramming of resident cells in the spiral ganglion into PANs. To the best of our knowledge, other than the reprogramming of non-sensory epithelial cells into induced neurons (iN) from our group (Puligilla et al., 2010; Nishimura et al., 2014) and our recent reprogramming of neonatal glial cells (Noda et al., 2018) there have been no other attempts at direct reprogramming in the peripheral auditory system (PAS). This review will herein summarize the historical perspectives and recent advances made in direct reprogramming, within the context of regenerative medicine, to propose this strategy as a novel intervention for the treatment of hearing loss. As a second objective, this review aims to position the PAS as an informative model for the study of regenerative medicine both *in vitro* and *in vivo*.

## Glia Within the Inner Ear Spiral Ganglion Offer an Advantageous Source for Direct Reprogramming

It is important to consider the target cell type for direct reprogramming since cells acquire lineage specific epigenetic markers during development (Ho and Crabtree, 2010; Vierbuchen and Wernig, 2012). These genetic signatures may partially explain why it is apparently more difficult to transdifferentiate distantly related lineages (Vierbuchen et al., 2010). Glial cells were first found to be easily converted into neuron-resembling cells through expression of a small number of transcription factors including *Pax6* alone (Heins et al., 2002) or *Neurog2* and *Ascl1* (Berninger et al., 2007). Subsequently, other combinations of transcription factors, such as *Brn2, Ascl1*, and *Myt1l* (Vierbuchen et al., 2010) or even *Ascl1* alone (Chanda et al., 2014), were found to be able to convert more distant cell types into neurons. These data indicated that it was possible to coax cells to become a cell type with a very different history using only a few, or even one, transcription factor(s). However, it appeared that iNs produced by fibroblasts take longer to mature than glial-derived iNs, presumably due to additional stages required in converting cells from a more distant lineage (Berninger et al., 2007; Heinrich et al., 2011; Wapinski et al., 2013; Chanda et al., 2014). If there are, in-fact, distinct stages involved, at least theoretically, it would be easier for glia to progress through these switches in state since both neurons and glia in the CNS naturally derive from the same population of neural progenitor cells (Bertrand et al., 2002). In fact, mutations in *Ascl1* and *Neurog2* result in premature development of astrocytic precursors instead of neural precursors, and expression of *Ascl1* both simultaneously commits progenitors to a neural fate and inhibits the glial developmental program (Bertrand et al., 2002). Adult pools of neural progenitor cells in the subventricular zone and hippocampal subgranular zone, which express *Ascl1*, also display glial characteristics and radial glia are a source of neurons during development (Malatesta et al., 2000; Doetsch, 2003; Kriegstein and Alvarez-Buylla, 2009). In the zebrafish retina, Müller glia act as a population of latent neural stem cells that can be activated after lesion to replace retinal neurons (Raymond et al., 2006). This process is dependent on upregulation of *Ascl1* (Ramachandran et al., 2010), indicating that normal processes of development and repair from damage can force glial-like cells to undergo transdifferentiation into neurons. Unfortunately, in the PAS no analog exists; however, multipotent stem cells have been discovered within the inner ear; in the utricle (Li et al., 2003) and in the spiral ganglion (Oshima et al., 2007; Zhang et al., 2011; Diensthuber et al., 2014; Li et al., 2016; McLean et al., 2016). These cells have the potential to form neurites, develop synapses and express neuronal markers *in vitro*, but it is unclear whether they naturally repopulate the spiral ganglion post-injury (Li et al., 2003, 2016). Nevertheless, given the similar history and location of glia in the spiral ganglion these cells likely have the highest conversion potential in regenerating auditory neurons to restore hearing. In fact, we have recently published an analysis of the transcriptome upon neuronal induction of spiral ganglion glial cells where we observed a marked upregulation of key neuronal signatures and downregulation of key glial signatures, indicating the high potential of reprogramming glial cells into neurons (Noda et al., 2018).

Glia are the support cells of the nervous system. They comprise at least 50% of the cells in the brain and 80% of the cells in the peripheral nerves (Rowitch and Kriegstein, 2010; Zuchero and Barres, 2015). In the brain, macroglia are derived from the same precursors as neurons. Early in development neuroepithelial progenitor cells differentiate into radial glia and these cells are converted first into neurons and then into astrocytes and oligodendrocytes (Malatesta et al., 2000). In the PAS on the other hand, glia and sensory neurons arise from different embryonic sources, the neural crest and the otic placode, respectively (D’Amico-Martel and Noden, 1983; Sandell et al., 2014). These migratory neural crest cells and neural precursors work in tandem during morphogenesis for the proper development of the cochleovestibular nerve (Sandell et al., 2014). In the spiral ganglion, the two major types of glia are satellite cells, which populate the area surrounding the cell bodies of sensory neurons, and Schwann cells, which migrate toward axonal projections (**Figure 1**) (Zuchero and Barres, 2015). Glia promote neuronal survival, provide nutrients and metabolic support, remove and recycle neurotransmitters, shape synapses, and form myelin sheaths (reviewed in Zuchero and Barres, 2015). In the CNS, astrocytes are additionally critical in regulating blood flow and in forming the blood–brain barrier. Although, the cochlea is similarly protected by a blood-labyrinth barrier, resident glial cells do not appear to be involved (Shi, 2016). Glia are also critical in the response to neural injury and disease. In the CNS, damage caused by acute injury, infection, ischemia and neurodegeneration results in an intricate balance between inflammation, cell death and debris removal (reviewed in Burda and Sofroniew, 2014). One hallmark feature of CNS insult is the proliferation of astrocytes. This process, known as reactive gliosis, results in the formation of a glial scar that prevents the spread of inflammation and protects viable cells (Faulkner et al., 2004). Unfortunately, recent studies have suggested that some reactive glia may play an emerging role in neurotoxicity (Liddelow et al., 2017; Qian et al., 2017) and old glial scars are also believed to inhibit axonal regeneration both physically and chemically through the release of extracellular matrix products (Kimura-Kuroda et al., 2010). Interestingly, there is alternative evidence to suggest that reactive gliosis may be involved in directing uncommitted cells toward a neurogenic fate (Robel et al., 2011). Alternatively, the increased incidence of transdifferentiation following reactive gliosis might instead be related to the post-injury environment since reprogramming experiments are similarly found to be more successful when induced after injury (Heinrich et al., 2014; Chiche et al., 2016; Mosteiro et al., 2016). The post-injury environment is associated with the release of inflammatory cytokines, which in-turn are responsible for several reactive processes such as triggering glial scarring and activating endogenous neural stem cells (Alvarez-Buylla and Garcia-Verdugo, 2002; Arvidsson et al., 2002; Yamashita et al., 2006; Chen et al., 2017). Hence, reprogramming glia may be useful for eliminating, or at least shrinking, glial scars by converting these cells into neurons.

In the PAS, damage can be caused directly to auditory neurons or indirectly through the loss of hair cells (Kujawa and Liberman, 2015). Reminiscent of reactive gliosis in the CNS, in the immediate period following injury there is marked proliferation of glial cells expressing *Sox2* (Lang et al., 2011, 2015). These *Sox2*-expressing glial cells display characteristics similar to neural progenitor cells, comparable to the neurogenic cells found after injury in the CNS (Lang et al., 2015). Despite the fact that neural stem cell niches are found in the spiral ganglion and cranial nerve VIII, unlike the CNS there is no evidence to suggest that there is recovery of neurons after damage (Oshima et al., 2007; Zhang et al., 2011; Diensthuber et al., 2014; Li et al., 2016). Perhaps these cells in the spiral ganglion play more of a neuroprotective role rather than replacing lost neurons. It is also not clear whether the PAS equivalent of reactive gliosis occurs after primary degeneration of PANs or if it only occurs after injury. Additionally, there is some overlap between the two systems since astrocytes and Schwann cells may migrate across the peripheral and central nervous system transitional zone following damage along the cochlear nerve (Hu et al., 2014). Elsewhere in the PNS, neurons have been found to retain some regenerative capacity, a feature likely related to glial interplay since Schwann cell dysfunction in age is thought to play a role in limiting regeneration (Painter, 2017). In sum, glia provide a similar role in both the CNS and PAS. They present similar challenges for attempts to regenerate lost neurons, and could provide significant advantages in that success in one field could lead to translatable results in the other.

## *In Vitro* Neuronal Reprogramming and Cellular Transplantation

It was previously thought that somatic cells obeyed a strict program resulting in a static terminally differentiated state. However, recently it has become accepted that cells are not locked into a certain state but are amenable to changing conditions (Sieweke, 2015). In fact, transcription factors can remodel cells into other differentiated cell types, a technique known as direct reprogramming. This is a beneficial strategy since it can bypass the lengthy induced pluripotent stem cell (iPSC) stage and also consequently decrease the chance of tumorigenesis due to latent pluripotent cells (Kelaini et al., 2014). Reprogramming has had major success in converting various cell types into others; including pancreatic β cell islets (Zhou et al., 2008), brown adipose tissue (Kajimura et al., 2009), cardiomyocytes (Ieda et al., 2010), and neurons (Vierbuchen et al., 2010; Pang et al., 2011). Most of this work has been performed using a combination of transcription factors, miRNAs and small chemical compounds *in vitro* (Li et al., 2015; Cao et al., 2016; Masserdotti et al., 2016; Gao et al., 2017). Using these techniques, it has become theoretically possible to generate new tissue and potentially even organs from an individual’s own cells with reduced tumourigenic side effects.

*In vitro* transdifferentiated cells can be used for the autologous transplantation of tissues, organs or cells. The earliest experiments demonstrating the transplantation of iNs to the CNS used transcription factor-based reprogramming to induce dopaminergic neurons from fibroblasts for the treatment of Parkinson’s disease. These cells were able to successfully integrate into the nervous circuit (Caiazzo et al., 2011), and even resulted in some functional recovery in an animal model of Parkinson’s disease (Kim et al., 2011). In the PAS, the only study to-date involving *in vitro* differentiated iNs and subsequent transplantation relied on a directed differentiation protocol to convert human iPSCs into glutamatergic neurons to be transplanted into guinea pig inner ears (Ishikawa et al., 2017). On a histological level these cells were incorporated into Rosenthal’s canal, but circuit integration and recovery of auditory function were not assessed and the number of cells remaining after 2 weeks was significantly reduced, presumably due to the host system’s immune response (Ishikawa et al., 2017). Other cases of transplantation in the spiral ganglion have used ESCs (Coleman et al., 2006) and iPSCs (Nishimura et al., 2009) or neurons extracted from other sources, such as embryonic dorsal root ganglion neurons (Hu et al., 2005). These studies demonstrated that it was possible for ESCs or iPSCs to differentiate into glutamatergic iNs that could form synapses with cochlear hair cells and could survive up to 4 weeks. However, these studies did not test recovery of auditory function or survival after a longer period.

Others have instead generated induced neural stem cells (iNSCs) *in vitro* for transplantation and differentiation *in vivo*. In the CNS, researchers have generated iNSCs by overexpressing some of the Yamanaka pluripotency factors and neuron-related transcription factors, such as *Brn2*, along with exposure to small molecules (Kim et al., 2011; Lujan et al., 2012). Ring et al. (2012) were able to directly generate iNSCs from fibroblasts using *Sox2* alone (Ring et al., 2012). These Sox2 derived iNSCs were able to differentiate into various mature neuronal and glial subtypes when transplanted in the mouse brain (Ring et al., 2012). Similarly, Lee et al. (2015) were able to differentiate blood cell derived iNSCs using GSK3 and SMAD inhibitors along with *Oct4* overexpression into dopaminergic and nociceptive neurons when transplanted *in vivo* (Lee et al., 2015). Kim et al. (2014) could generate induced neural crest-like cells that could be differentiated into peripheral neurons and glia by overexpression of *Sox10* when paired with canonical Wnt activation (Kim et al., 2014). In the PAS, a handful of studies have indicated that the differentiation of progenitor or stem cells toward auditory neurons is a similarly promising strategy. iNs using this method have been shown to abundantly repopulate the auditory nerve and send extensions toward the sensory epithelium (Corrales et al., 2006; Shi et al., 2007). Hu et al. (2017) thoroughly analyzed neural stem cell derived neurons and discovered they were able to form functional synapses with cochlear nucleus neurons *in vitro* (Hu et al., 2017). Chen et al. (2012), using otic progenitors derived from hESCs, discovered that it was possible to restore some auditory function after transplantation (Chen et al., 2012), and Song et al. (2017) found that acquisition of neuronal properties from otic progenitors could be accelerated upon *Neurog1* overexpression (Song et al., 2017). Hackelberg et al. (2017) similarly observed integration of differentiated neurons from human-derived neural progenitor cells when implanted into the guinea pig internal auditory meatus after induced auditory neuropathy (Hackelberg et al., 2017). To improve growth of neurites toward PAN targets, Hackelberg et al. (2017) simultaneously delivered a nanofibrous scaffold. However, these studies implanted cells only shortly after induced auditory neuropathy, hence the differentiation of iNs may be the result of an early post-injury environment. This temporary niche is supplied with growth factors and cytokines not normally present and has the potential to even stimulate ESCs transplanted at the internal auditory meatus portion of the auditory nerve to migrate toward Rosenthal’s canal and the scala media (Sekiya et al., 2006). Therefore, it is possible that this environment may have a profound impact on transplantation with vastly different results than a late-injury model of auditory neuropathy since this environment is often inhibitory (Lang et al., 2008). These approaches that generate multipotent precursors (e.g., iNSCs, otic progenitors) are useful because these cells are expandable, they have a reduced potency such that they can only differentiate into a limited number of cell types, and are amenable to the environmental cues in the transplanted setting. However, the proliferative capability of these cells is still of concern.

Despite the usefulness of *in vitro* reprogrammed cells, there are major limitations to transplantation. Cellular transplantation is an invasive process that can result in death of both cells from the original tissue and the transplanted ones. Therefore, cellular transplantation requires tremendous numbers of cells to maximize the yield of viable cells that integrate into host tissues. Fortunately, Rigamonti et al. (2016) have recently developed a large-scale production method to differentiate iPSCs into mature cortical or motor neurons using a suspension culture system (Rigamonti et al., 2016). These iNs could form integrated neural networks and generate synchronized action potentials within the culture system, thereby addressing the need to create large amounts of iNs. However, a second obstacle for transplantation efforts using *in vitro* differentiated cells is immunogenicity. A characteristic of iPSCs and ESCs maintained in culture for long periods of time is the development of aberrant surface proteins which are passed onto differentiated cells and trigger the immune system (as reviewed in Tang and Drukker, 2011). This problem is supposedly due to the incomplete conversion of cells *in vitro* and is not observed with *in vivo* reprogramming (Tang and Drukker, 2011). Hence the completeness of conversion may be related to extrinsic factors provided to cells within the *in vivo* cellular niche. This consequence of ectopically transplanted cells is also dependent on cell type, since it does not always result in an immune response (Tapia and Schöler, 2016). In sum, *in vitro* lineage conversion is advantageous for understanding the molecular features of transdifferentiation; however, several difficult obstacles for the transplantation of *in vitro* derived cells limits its usefulness as a therapy for humans. A more promising solution that avoids some of these issues is the *in vivo* reprogramming of spiral ganglion glia into neurons.

## *In Vivo* Reprogramming of Glia and Functional Studies

**FIGURE 2:**
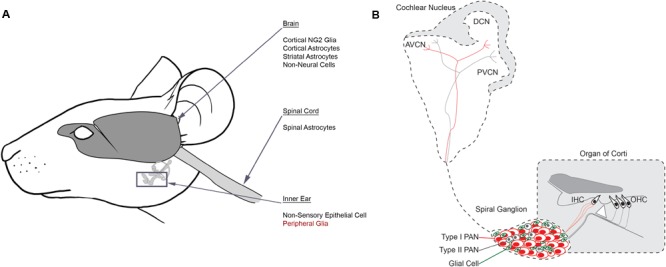
Reprogramming glial cells into neurons *in vivo*. **(A)** Schematic identifying source cell populations that have been converted into induced neurons either *in vitro* or *in vivo*. Induced neurons have been derived from several distinct cell populations in the brain, spinal cord and inner ear. Many of these cells include glial cell types. Peripheral glia (red) are suggested targets for future reprogramming efforts in the inner ear. **(B)** Schematic identifying the auditory circuit leading from the organ of Corti within the inner ear, through the spiral ganglion and ultimately to the cochlear nucleus within the brainstem. Type I PANs (red) form multiple synapses with inner hair cells (IHC) and Type II PANs (gray) innervate outer hair cells (OHC). Glia (green) are interspersed with PANs in the spiral ganglion. Afferent fibers from both PAN subtypes project to the cochlear nucleus based on their unique characteristics, establishing a tonotopic map. AVCN, anterior ventral cochlear nucleus; DCN, dorsal cochlear nucleus; PVCN, posterior ventral cochlear nucleus.

*In vivo* reprogramming refers to cellular reprogramming that takes place within a living organism through direct intervention methods such as gene therapy. *In vivo* reprogramming takes advantage of the microenvironments that already exist in the body and bypasses some of the complications associated with cell grafting. As an added benefit, *in vivo* reprogramming is perhaps more efficient than *in vitro* reprogramming since *in vivo* strategies (Qian et al., 2012; Liu et al., 2015) appear to be more successful in converting cardiomyocytes and neurons than *in vitro* strategies (Ieda et al., 2010; Vierbuchen et al., 2010; Pang et al., 2011; Heinrich et al., 2015). Attempts at reprogramming glia into neurons *in vivo* have largely focused on two major strategies: converting glia into neuroblasts and differentiating these cells into iNs or directly converting glia into iNs (Smith and Zhang, 2015; Smith et al., 2016, 2017) (**Figure 2A** and **Table 1**).

**Table 1:** Summary of *in vivo* reprogramming strategies in the nervous system.

Cell type	Reprogramming factor(s)	Generated cells	Location of reprogramming	Functional study	Reference
**Brain**					
Astrocyte	Ascl1 + Brn2 + Myt1l	NeuN+ neurons	Striatum		Torper et al., 2013
Astrocyte	Sox2 (+ BDNF + Noggin)	NeuN+ neurons	Striatum	Spontaneous synaptic currents.	Niu et al., 2013
Reactive astrocyte	Neurog2 (+ FGF + EGF)	Glutamatergic neurons/Glutamatergic and GABAergic neurons	Cortex/striatum		Grande et al., 2013
Reactive astrocyte	NeuroD1	Glutamatergic neurons	Cortex	Spontaneous and evoked synaptic responses.	Guo et al., 2014
NG2 glia	NeuroD1	Glutamatergic and GABAergic neurons	Cortex	Spontaneous and evoked synaptic responses.	Guo et al., 2014
NG2 glia	Sox2	GABAergic	Cortex	Spontaneous synaptic currents.	Heinrich et al., 2014
Astrocyte	Ascl1	Glutamatergic and GABAergic neurons	Striatum	Spontaneous and evoked synaptic responses.	Liu et al., 2015
NG2 glia	Ascl1 + Lmx1a + Nurr1	Glutamatergic and GABAergic neurons	Striatum	Spontaneous and evoked synaptic responses.	Torper et al., 2015
Astrocyte	Neurog2 + Bcl2	Glutamatergic pyramidal neurons	Cortex		Gascón et al., 2016
Astrocyte	NeuroD1	NeuN+ neurons	Cortex/striatum		Brulet et al., 2017
Astrocyte	NeuroD1 + Ascl1 + Lmx1a + miR218	Dopaminergic neurons	Striatum	Spontaneous and evoked synaptic responses. Rescues behavior in Parkinson’s model.	di Val Cervo et al., 2017
Astrocyte	Ascl1 + Pitx3 + Lmx1a + Nurr1 ( + gold nanoparticles and electromagnetic field exposure)	Dopaminergic neurons	Striatum	Spontaneous and evoked synaptic responses. Rescues behavior in two different Parkinson’s models.	Yoo et al., 2017
**Spinal Cord**					
Astrocyte	Sox2 ( + VPA)	GABAergic			Su et al., 2014
Astrocyte	Sox2 ( + VPA + BDNF + Noggin)	Primarily glutamatergic (GABAergic, glycinergic, serotonergic, cholinergic)			Wang et al., 2016

Neuroblasts are the expandable precursors to neurons, hence by converting resident glial cells into neuroblasts it is possible to increase the number of cells while simultaneously creating new neurons *in vivo*. The generation of neuroblasts *in vivo* can be achieved by the ectopic expression of the *Sox2* transcription factor, both in the brain (Niu et al., 2013, 2015) and in the spinal cord (Su et al., 2014; Wang et al., 2016). When animals concurrently overexpress neurotrophic factors such as BDNF and noggin or are orally administered the histone deacetylase inhibitor valproic acid, these neuroblasts are found to differentiate into iNs (Niu et al., 2013, 2015; Su et al., 2014). This method of creating iNs through a multipotent neuroblast intermediate involves guiding glia through distinct cell stages. Transduced cells first become neuroprogenitor cells that express *Ascl1*. They develop into *Doublecortin* expressing neuroblasts and commit to a neuronal fate (Niu et al., 2015). They then mature when supplied with exogenous neurotrophic factors. iNs produced from this step-wise differentiation protocol using *Sox2* could also reliably generate action potentials and form synapses with endogenous neurons. This technique, which both increases the number of source cells while creating functional neurons can be useful for neuronal regeneration approaches. It is unclear if *Sox2* could drive the conversion of peripheral glia into neuroblasts since the upregulation of *Sox2* is a characteristic response after injury in the PAS for glial proliferation (Lang et al., 2011). Additionally, reactive gliosis typically results in extensive amounts of proliferating glia, hence this strategy may not be necessary to create sufficient numbers of iNs in the inner ear. However, reprogramming glia to neuroblasts remains an option if direct conversion in the spiral ganglion yields uncharacteristic low numbers of iNs or there are too few source cells remaining in the spiral ganglion, as seen in older animals (Keithley et al., 1989).

Many other researchers have used neurogenic transcription factors to reprogram glial cells directly into iNs. The overexpression of *Ascl1, Brn2*, and *Myt1l* converted parenchymal astrocytes into neurons in the adult mouse striatum (Torper et al., 2013). Similar to the *in vitro* studies on neuronal reprogramming, *Ascl1* on its own also converted midbrain astrocytes (Liu et al., 2015) and reactive astrocytes from the subventricular zone (Faiz et al., 2015) into functional neurons. *NeuroD1* alone was also found to convert astrocytes into mature neurons (Guo et al., 2014; Brulet et al., 2017). Aside from astrocytes, NG2 glia have been targeted as a potential source cell type. NG2 glia are the precursors to oligodendrocytes and could be converted into iNs by *NeuroD1* (Guo et al., 2014), *Sox2* (Heinrich et al., 2014) or the combination of *Ascl1, Lmx1a*, and *Nurr1* (Torper et al., 2015). In a cortical injury mouse model, *Neurog2* and the addition of growth factors to non-neural cortical cells was sufficient for cells to adopt a neuronal fate (Grande et al., 2013). Interestingly, different areas of the brain appeared to have characteristically different responses. *Neurog2-*transfected cells in the striatum reliably developed into both glutamatergic and GABAergic iNs, whereas cells in the neocortex only developed into glutamatergic iNs. This difference in reprogramming suggests that local environmental cues can have a considerable effect on the outcome of conversion. Alternatively, this may be the result of a developmental effect. Cells may become regionally primed toward neighboring neural subtypes through the process of development and this phenotypic preference materializes during reprogramming (Liu et al., 2015; Masserdotti et al., 2015; Chouchane et al., 2017). Regardless of the mechanism, unfortunately, both areas regenerated less than 5% of the number of neurons lost from injury. Gascón et al. (2016) hypothesized that the low yields achieved after cellular reprogramming were the result of a switch in metabolism from aerobic respiration in glia to anaerobic respiration in neurons, and the failure to transition resulted in cellular death (Gascón et al., 2016). Hence, they combined the expression of *Neurog2* with *Bcl2*, an anti-apoptotic transcription factor, to increase iN yields from astrocytes. Instead of the predicted apoptotic pathway, *Bcl2* appeared to aid in reprogramming by reducing lipid peroxidation, a marker of ferroptosis (Gascón et al., 2016). Mosteiro et al. (2016) on the other hand showed that cells failing to reprogram undergo senescence and secrete cytokines that actually facilitate the reprogramming of other cells (Mosteiro et al., 2016). They found that by using a *Bcl2* inhibitor they could selectively kill senescent cells and consequently decrease reprogramming efficiency (Mosteiro et al., 2016). From the results of Gascón et al. (2016) and Mosteiro et al. (2016) it is evident that cells transfected with reprogramming factors can fail to reprogram and instead enter an alternative pathway, whether that may be ferroptosis or senescence. However, it is not clear why some cells are successful at reprogramming whereas others are interrupted along the way. These studies are part of an emerging development in the reprogramming field to understand the molecular roadblocks that prevent conversion in hopes of facilitating reprogramming instead of inundating cells with neurogenic transcription factors. These examples that newly derived iNs from a variety of sources could form functional connections with endogenous neurons when reprogrammed *in vivo* are exciting developments, but further studies on the mechanisms preventing reprogramming are critical for developing strategies that can be efficient solutions for neurodegenerative diseases.

In studies of neural regeneration in the CNS, damage paradigms involve the use of transgenic mice or targeted lesions. This is a beneficial approach for diseases tied to a specific phenotype or pathology but not when the genetics are unknown and/or broad. In the PAS this problem is circumvented since it is possible to abolish hearing by specifically targeting the destruction of PANs through the use of the chemical ouabain (Yuan et al., 2014). The amount and delivery of ouabain is particularly important, because at higher concentrations it can also influence hair cells (Fu et al., 2012). Nevertheless, this method of selectively destroying endogenous PANs allows researchers to specifically focus on regeneration of neurons and hearing instead of other symptomatic effects. An additional advantage to reprogramming PAS glia into neurons is the relative homogeneity of PANs compared to the innumerable subtypes of neurons found in the CNS. In addition to simply generating neurons for regenerative medicine, it is also critical to differentiate these cells into the required subtype(s). Fortunately, based on studies in the CNS most astrocytes induced to convert *in vitro* have been found to retain regional specification consistent with the location where glial cells were derived. This leads to the corresponding creation of GABAergic neurons in the cortex (Masserdotti et al., 2015), and both GABAergic and glutamatergic neurons in the midbrain (Liu et al., 2015). The neural subtypes formed when spiral ganglion glial cells are converted has yet to be examined; however, based on the work completed in the CNS it is likely that these cells will become glutamatergic neurons, which is consistent with the neuronal subtype of PANs (Reijntjes and Pyott, 2016). In fact, spiral ganglion derived neural stem cells almost exclusively differentiate into spiral ganglion-like glutamatergic cells (Li et al., 2016). Fortunately for the purposes of reprogramming in the inner ear this strategy should be sufficient to restore hearing since all PANs are glutamatergic neurons. Therefore, the *in vivo* strategies already succeeding in the brain can be applied to the PAS as-is without need for refinement of neural subtype.

Additionally, reprogramming in the PAS is advantageous since there are already well-established methods that can be easily implemented to robustly validate the integration of reprogrammed iNs into pre-existing circuits. These types of rigorous functional studies are critical to ensure that iNs are working as intended and rescuing the impaired phenotype rather than simply adding cells. Functional studies of reprogramming in the CNS can be difficult since many neurodegenerative conditions involve widespread damage, such as in Alzheimer’s disease, and thus require iNs to form extensive connections with endogenous neurons in far-reaching areas of the brain (Goldman, 2016). This is not to mention the sheer number of iNs that would be required to rescue the phenotype of Alzheimer’s disease. Given our current state of technology, it is not clear how to both broadly reprogram glia in the brain and prevent off-target reprogramming elsewhere in the body. On the other hand, diseases like Parkinson’s and Huntington’s where lost neurons are restricted to a single phenotype and/or location may benefit from the reprogramming techniques currently available. There is evidence of some motor rescue in humans with Parkinson’s disease when grafted with fetal dopaminergic tissue (Cicchetti et al., 2009; Barker et al., 2015), but this does not lead to stable recovery and typically results in dyskinesias. The instability of grafted tissue may be related to heterologous transplantation since dopaminergic neurons derived from autologous iPSCs *in vitro* can stably reinnervate the host brain and rescue some motor function when implanted in non-human primates (Hallett et al., 2015). Recently, two breakthrough studies have shown that striatal astrocytes can be reprogrammed into dopaminergic neurons *in vivo*. These induced dopaminergic neurons could reliably generate action potentials and rescue some motor behavior in mouse models of Parkinson’s disease (di Val Cervo et al., 2017; Yoo et al., 2017). These studies used a combinations of familiar transcription factors and/or microRNAs (**Table 1**). In a unique approach, Yoo et al. (2017) also supplemented gene delivery in the mouse striatum with gold nanoparticles that were affected by an electromagnetic field for 3 weeks (Yoo et al., 2017). Stimulation by an electromagnetic field was thought to increase expression of proteins that influenced the chromatin state, thus robustly activating neuronal genes. Previously only glutamatergic or GABAergic neurons had been created *in vivo*, hence this elusive feat demonstrated by two labs simultaneously indicates the tremendous innovation happening in the field of regenerative medicine. In comparison to the brain, the spiral ganglion in the PAS is a physically small and restricted niche that is separated from the rest of the body by the blood-labyrinth barrier and is composed of only glutamatergic neurons. Although, PANs only form connections at two ends, with the hair cells of the cochlea and the neurons of the cochlear nucleus of the brain, these cells form networks in a precisely organized tonotopic layout (Appler and Goodrich, 2011). Further complexity is added when considering spontaneous discharge rate, activation threshold, and sound intensity coding of PANs, which inform the termination patterns of PANs in the cochlear nucleus (Kawase and Liberman, 1992). However, functional analyses of the PAS can be relatively easily evaluated using objective audiometric tests. The auditory brainstem response (ABR), is a non-invasive recording of electrical activity transmitted between cranial nerve eight and the brainstem (Davies, 2016). It is logged using electrodes placed on the surface of the scalp (Guo et al., 2014). ABR waveforms have a distinctive five wave pattern that can be used to identify the location of pathology, and therefore can be used to test integration of iNs into the auditory circuit. In the case that an auditory evoked potential cannot be detected by the ABR it is possible to use electrically evoked compound action potentials to test the electrical activity of the auditory nerve independent of auditory activity (Ramekers et al., 2015). Although this is an invasive strategy that requires implanting electrodes in the cochlea and brain, it can be useful to test whether iNs are electrically active but suffer from functional connectivity between the cochlea and cochlear nucleus. In either case, reprogramming in the PAS can be robustly tested using powerful audiometric techniques. These features make the PAS an attractive opportunity to examine reprogramming techniques on a smaller scale with equally landmark implications as studies in the CNS.

## Challenges and Future Directions

Building upon these foundational studies on direct neuronal reprogramming of glia in the CNS, the direct reprogramming of spiral ganglion glial cells into PANs in the PAS appears likely to be a feasible strategy to restore hearing. Ample *in vitro* and *in vivo* evidence indicate that glia are amenable to conversion into functional neurons. In terms of delivery to the spiral ganglion, proneurogenic genes can be administered using adeno-associated viruses since they have low toxicity and immunogenicity while being safe for human usage (Mueller and Flotte, 2008). However, for strategies such as this to be useful, the success of conversion in aged mice will need to be tested since the most likely recipient for regenerative medicine efforts will be adults. Ahlenius et al. (2016) have shown that cells acquired from older mice display senescence, overexpress the transcription factor *Foxo3* and are more difficult to reprogram *in vitro* (Ahlenius et al., 2016). Directly reprogrammed neurons additionally retain age-related signatures, which may include nucleocytoplasmic defects that can critically alter the cellular phenotype *in vitro*, although this has not yet been observed *in vivo* (Mertens et al., 2015). On the other hand, Mosteiro et al. (2016) have shown that senescent cells secrete cytokines which increase the reprogramming efficiency of nearby cells when transduced with transcription factors *in vivo* (Mosteiro et al., 2016). Therefore, more research is needed on *in vivo* reprogramming in adult cells to elucidate the effectiveness of conversion on aged cells.

In the case that *in vivo* reprogramming can create suitable numbers of iNs, there is still the issue of forming functional synaptic connections with the mechanosensory hair cells of the cochlea and the brainstem. Given the nature of neural connections between the cochlea and the brainstem, it is likely that there will be equal or even greater success at reprogramming glia into neurons and circuit integration in the PAS than the CNS. This is because PANs are glutamatergic and have a single connection to the cochlea and another to the auditory center of the brain. A more difficult task in the PAS will be to establish tonotopic connections, which will be critical in restoring natural-like hearing (**Figure 2B**). It might be necessary to combine direct reprogramming with the delivery of neurotrophic factors through an osmotic pump (Sly et al., 2012) or a cell-based therapy (Zanin et al., 2014) to induce axon pathfinding and synapse formation. Hence, more work will need to be done to see whether reliable neural connections are formed. If successful, the PAS has the potential to become a model system to test regenerative medicine approaches for many neurodegenerative diseases that would benefit from a gene therapy approach to cell regeneration.

## Author Contributions

SM, C-LZ, and AD: conceptualization and writing. AD: supervision and funding.

## Conflict of Interest Statement

The authors declare that the research was conducted in the absence of any commercial or financial relationships that could be construed as a potential conflict of interest.
